# N-Terminal Phosphorylation of the Dopamine Transporter Is Required for Amphetamine-Induced Efflux

**DOI:** 10.1371/journal.pbio.0020078

**Published:** 2004-03-16

**Authors:** Habibeh Khoshbouei, Namita Sen, Bipasha Guptaroy, L'Aurelle Johnson, David Lund, Margaret E Gnegy, Aurelio Galli, Jonathan A Javitch

**Affiliations:** **1**Department of Molecular Physiology and Biophysics and Center for Molecular Neuroscience, Vanderbilt UniversityNashville, TennesseeUnited States of America; **2**Center for Molecular Recognition, Columbia UniversityNew York, New YorkUnited States of America; **3**Department of Pharmacology, University of MichiganAnn Arbor, MichiganUnited States of America; **4**Departments of Psychiatry and Pharmacology, College of Physicians and SurgeonsColumbia University, New York, New YorkUnited States of America

## Abstract

Amphetamine (AMPH) elicits its behavioral effects by acting on the dopamine (DA) transporter (DAT) to induce DA efflux into the synaptic cleft. We previously demonstrated that a human DAT construct in which the first 22 amino acids were truncated was not phosphorylated by activation of protein kinase C, in contrast to wild-type (WT) DAT, which was phosphorylated. Nonetheless, in all functions tested to date, which include uptake, inhibitor binding, oligomerization, and redistribution away from the cell surface in response to protein kinase C activation, the truncated DAT was indistinguishable from the full-length WT DAT. Here, however, we show that in HEK-293 cells stably expressing an N-terminal-truncated DAT (del-22 DAT), AMPH-induced DA efflux is reduced by approximately 80%, whether measured by superfusion of a population of cells or by amperometry combined with the patch-clamp technique in the whole cell configuration. We further demonstrate in a full-length DAT construct that simultaneous mutation of the five N-terminal serine residues to alanine (S/A) produces the same phenotype as del-22—normal uptake but dramatically impaired efflux. In contrast, simultaneous mutation of these same five serines to aspartate (S/D) to simulate phosphorylation results in normal AMPH-induced DA efflux and uptake. In the S/A background, the single mutation to Asp of residue 7 or residue 12 restored a significant fraction of WT efflux, whereas mutation to Asp of residues 2, 4, or 13 was without significant effect on efflux. We propose that phosphorylation of one or more serines in the N-terminus of human DAT, most likely Ser7 or Ser12, is essential for AMPH-induced DAT-mediated DA efflux. Quite surprisingly, N-terminal phosphorylation shifts DAT from a “reluctant” state to a “willing” state for AMPH-induced DA efflux, without affecting inward transport. These data raise the therapeutic possibility of interfering selectively with AMPH-induced DA efflux without altering physiological DA uptake.

## Abstract 2

Using mutated dopamine transporters, the authors find that they can separate the actions of amphetamine from the normal function of the dopamine transporter

## Introduction

The dopamine transporter (DAT) plays a critical role in the synaptic clearance of dopamine (DA) by mediating the reuptake of DA released into the presynaptic terminal (Amara and Kuhar 1993; Giros and Caron 1993). It thereby regulates the strength and duration of the dopaminergic response. DAT is also the site of action of several psycho-stimulant drugs, including amphetamine (AMPH) and cocaine (Kuhar et al. 1991). As a substrate, AMPH competitively inhibits DA reuptake, thereby increasing synaptic DA concentration and enhancing the rewarding property of the dopaminergic system. Additionally, AMPH elicits the release of DA through the transporter in the brain (Fischer and Cho 1979; Jones et al. 1998) and in heterologous cells expressing DAT (Eshleman et al. 1994; Wall et al. 1995; Sitte et al. 1998). AMPH-induced DA efflux is thought to be mediated by a facilitated exchange diffusion process, in which inward transport of substrates increases the availability of inward-facing binding sites of the transporter (Fischer and Cho 1979), which leads thereby to increased efflux of cytosolic substrates. Emerging evidence, however, indicates that inward and outward transport of monoamines may differ in more fundamental ways. In particular, it appears that AMPH-induced DA efflux does not rely exclusively on the ability of AMPH to increase the availability of inward-facing DATs (Chen and Justice 2000) but also relates to the ability of AMPH to induce uncoupled currents (Sitte et al. 1998) and to increase intracellular sodium (Khoshbouei et al. 2003) and kinase activity (Kantor and Gnegy 1998). Although AMPH-induced currents have been shown to be of physiological relevance (Ingram et al. 2002), AMPH exerts its primary behavioral effects by inducing DA efflux (Wise and Bozarth 1987; Sulzer and Galli 2003). In addition, enhanced AMPH-induced DA efflux is associated with sensitization to repeated AMPH administration (Robinson and Becker 1986).

DAT is thought to comprise 12 transmembrane segments with cytoplasmic N-terminal and C-terminal domains (Giros and Caron 1993). There are numerous putative phosphorylation sites for various protein kinases in the intracellular domains (Giros and Caron 1993; Granas et al. 2003; Lin et al. 2003), and multiple protein kinases have been shown to regulate DAT function (Daniels and Amara 1999; Melikian and Buckley 1999; Granas et al. 2003). Treatment with AMPH also leads to increased intracellular accumulation of DAT (Saunders et al. 2000), and AMPH has been shown to increase striatal particulate PKC activity (Giambalvo 1992) through a calcium dependent pathway (Giambalvo 2003). Importantly, PKC activation leads to N-terminal phosphorylation of DAT in rat striatum (Foster et al. 2002). Consistent with this observation, we recently showed that deletion of the first 22 amino acids from DAT essentially eliminates^32^P incorporation into DAT in response to PKC activation (Granas et al. 2003). Surprisingly, this truncation did not affect PKC-induced internalization, thereby demonstrating that N-terminal phosphorylation of DAT is not essential for internalization. Since uptake, inhibitor binding, and oligomerization of this truncated DAT were also not significantly different from those of full-length DAT (Hastrup et al. 2001, 2003; Granas et al. 2003), N-terminal phosphorylation has not yet been associated with a functional effect.

PKC activation, however, has been shown to stimulate DAT-mediated release of DA (Davis and Patrick 1990; Giambalvo 1992; Kantor and Gnegy 1998). Moreover, AMPH-induced DA efflux is inhibited by the introduction of PKC inhibitors and by downregulation of PKC (Kantor and Gnegy 1998; Cowell et al. 2000; Kantor et al. 2001), whereas DA uptake is unaffected by these manipulations. This suggests that inward and outward transport can be independently regulated and led us to explore the hypothesis that N-terminal phosphorylation of DAT may be involved in AMPH-induced DA efflux. Here we report that deletion of the first 22 amino acids of DAT, as well as mutation of the five N-terminal serines to alanine, greatly decreases AMPH-induced DA efflux without affecting uptake. Mutation of these serines instead to aspartate, thereby mimicking phosphorylation, preserves efflux, suggesting that phosphorylation of one or more of these five N-terminal serines is essential for AMPH-induced DA release.

## Results/Discussion

**Figure 1:**
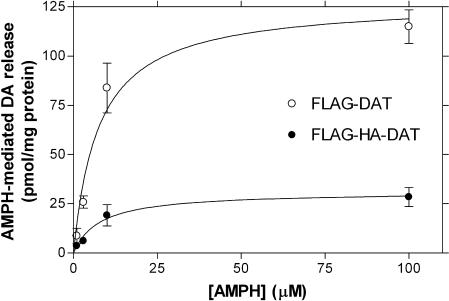
N-Terminal Truncation of DAT Impairs AMPH-Induced DA Efflux Cells were preloaded with 15 μM DA and superfused with AMPH at concentrations ranging from 1 to 100 μM. AMPH-induced DA efflux was defined as the amount of DA released in response to the given concentration of AMPH minus the baseline value. Baseline DA release did not differ between FLAG-HA-DAT and FLAG-DAT (13.2 ± 2.9 and 10.2 ± 1.8, respectively; *n =* 18). The V_max_ of efflux was 31.1 ± 4.6 and 128.3 ± 12.0 pmol/mg protein/fraction (F(2,27) = 52.6, *p* < 0.0001) with a K_m_ for amphetamine of 7.8 ± 4.1 and 7.6 ± 2.2 μM, for FLAG-HA-DAT and FLAG-DAT, respectively (*n* = 4). For [^3^H]DA uptake, the V_max_ was 15.4 ± 2.5 and 18.3 ± 2.2 pmol/min/mg protein with a K_m_ of 1.2 ± 0.8 and 1.1 ± 0.4 μM for FLAG-HA-DAT and FLAG-DAT, respectively (F(2,49) = 1.78, *p* > 0.17).

In our previous studies we created a mutant human DAT construct in which the first 22 amino acids were removed and replaced by tandem FLAG and HA epitope tags (FLAG-HA-DAT) (Hastrup et al. 2001, 2003). This construct was created to tag the protein and to remove Cys6 to facilitate biochemical studies. FLAG-HA-DAT expressed at wild-type (WT) levels in the plasma membrane, and we found it to be functionally normal in terms of uptake, inhibitor binding, DAT oligomerization, and PMA- and receptor-induced internalization FLAG-HA-DAT expressed at wilde-type (WT) levels in the plasma membrane, and we found it to be functionally normal in terms of uptake, inhibitor binding, DAT oligomerization, and PMA- and receptor-induced internalization (Hastrup et al. 2001, 2003; Granas et al. 2003). Since this construct lacks the first five serines in DAT (Ser2, Ser4, Ser7, Ser12, Ser13) and does not appear to be phosphorylated by PKC activation (Granas et al. 2003), we hypothesized that FLAG-HA-DAT might be impaired in AMPH-induced efflux. In accordance with this prediction, we found that AMPH-induced DA efflux was decreased by approximately 80% in the FLAG-HA construct relative to FLAG-tagged full-length DAT (FLAG-DAT) (Figure 1). This resulted from a decrease in the maximal rate of DA efflux and not from a change in the apparent affinity for AMPH in mediating efflux. In contrast, DA uptake by these two constructs was not significantly different (Figure 1, legend).

In a cell suspension (or in a population of adherent cells), it is difficult to assess the potential effects on efflux of a change in ionic gradients or membrane potential because the membrane potential and ionic gradients change freely depending on the stimuli. Indeed, AMPH has been shown to induce depolarization through a DAT-mediated uncoupled chloride conductance that can be gated by substrates such as AMPH (Ingram et al. 2002). Therefore, in order to quantify these effects under conditions where we could control the intracellular concentration of the substrates, DA, sodium, and chloride, as well as the membrane potential, we used amperometry in conjunction with the patch-clamp technique in the whole-cell configuration, a method that we have used previously to study the mechanism of efflux (Galli et al. 1998; Khoshbouei et al. 2003). We recorded DAT-mediated currents with the whole-cell pipette by stepping the membrane voltage from a holding potential of −20 mV to +100 mV while simultaneously measuring efflux as assessed by amperometric currents resulting from the release of DA. Consistent with our studies with cell populations, we found that AMPH-induced efflux was decreased at +100 mV by 91% ± 4% (*n* = 5) in FLAG-HA-DAT relative to FLAG-DAT. Surprisingly, the DAT-mediated whole-cell currents gated by AMPH, which have been shown to be uncoupled from the transport process (Sonders et al. 1997; Khoshbouei et al. 2003), were also reduced to a comparable extent (see below).

**Figure 2:**
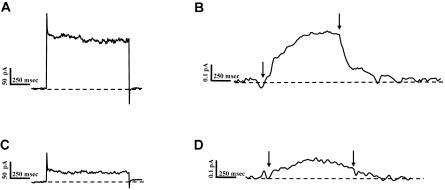
N-Terminal Truncation of DAT Reduces AMPH-Induced Currents and DA Efflux Cells were voltage clamped with a whole-cell patch pipette while an amperometric electrode was placed onto the cell membrane. The internal solution of the whole-cell patch pipette contained 2 mM DA. (A) Representative trace of AMPH-induced whole-cell current obtained from FLAG-DAT cells upon AMPH (10 μM) bath application. The membrane potential of the cell was stepped to +100mV from a holding potential of –20 mV. (B) Oxidation current acquired concomitantly to the whole-cell current represented in panel A. (C and D) Representative current traces (whole-cell and amperometric, respectively) obtained from FLAG-del22-DAT cells using the same experimental protocol as in (A) and (B).

This reduction in current and efflux resulted from the N-terminal deletion and not from the presence of the HA epitope, since a FLAG-tagged construct lacking the first 22 amino acids of DAT (FLAG-del22-DAT) but without any other added sequence showed a reduction in current and efflux similar to that of FLAG-HA-DAT. Figure 2 shows representative traces for the AMPH-induced current and DA efflux recorded at +100 mV obtained from FLAG-DAT (panels A and B, respectively) and FLAG-del22-DAT (panels C and D, respectively). In panels B and D, the upward (positive) deflections indicate DA oxidation and thus reflect DA efflux. At the onset of the voltage step, the amperometric electrode recorded an oxidative current (positive), which is indicative of DA efflux, and at the termination of the voltage step, the amperometric current relaxed to baseline. At +100 mV, the AMPH-induced whole-cell and oxidative currents recorded from FLAG-del22-DAT cells were much smaller than those recorded from FLAG-DAT cells: in FLAG-del22-DAT cells, the whole-cell currents were 21.8% ± 7.4% whereas the amperometric currents were 23.0% ± 2.5% of the equivalent currents recorded in FLAG-DAT cells (*n* = 5).

In marked contrast to this approximately 80% reduction, in the same two sets of stably transfected cells, the V_max_ for uptake of the substrate tyramine by FLAG-del22-DAT was 146% that by FLAG-DAT (Table 1). Neither the K_m_ for tyramine uptake (Table 1) nor the apparent K_i_ for inhibition of tyramine uptake by AMPH (37 ± 4 nM and 63 ± 18 nM, respectively; *n =* 5) or cocaine (214 ± 34 nM and 281 ± 33 nM, respectively; *n =* 4) was significantly different in FLAG-DAT and FLAG-del22-DAT. Cell-surface biotinylation studies revealed that the increased V_max_ in FLAG-del22-DAT was accounted for by an increased number of DAT molecules at the cell surface (Table 1) and suggested that the truncation had a minimal effect on the turnover rate of the transporter. These results are consistent with our previous studies on the FLAG-HA-DAT deletion construct expressed in EM4 cells, which also showed normal tyramine uptake (Hastrup et al. 2001), as well as with the DA uptake studies described above for FLAG-HA-DAT and FLAG-DAT expressed in HEK-293 cells (see Figure 1, legend).

**Table 1:**
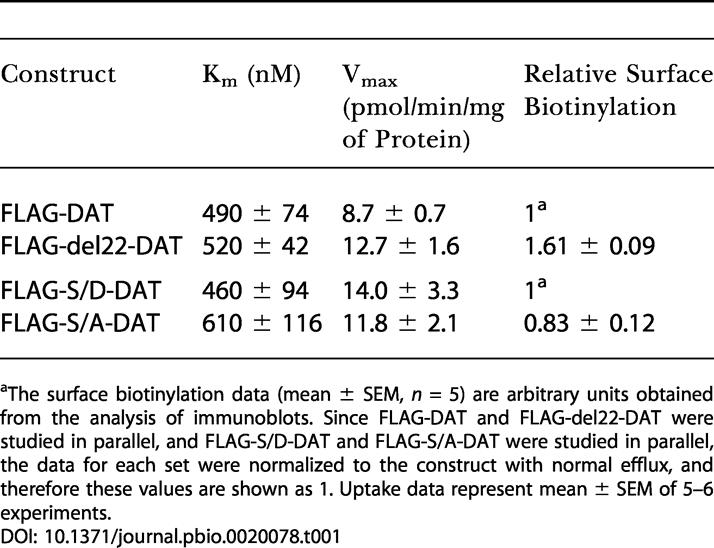
Kinetic Properties of [^3^H]Tyramine Uptake and Cell-Surface Localization of FLAG-DAT, FLAG-del22-DAT, FLAG-S/A-DAT, and FLAG-S/D-DAT

^a^The surface biotinylation data (mean ± SEM, *n* = 5) are arbitrary units obtained from the analysis of immunoblots. Since FLAG-DAT and FLAG-del22-DAT were studied in parallel, and FLAG-S/D-DAT and FLAG-S/A-DAT were studied in parallel, the data for each set were normalized to the construct with normal efflux, and therefore these values are shown as 1. Uptake data represent mean ± SEM of 5–6 experiments

If the reduction in the AMPH-induced current and efflux resulted from the loss of phosphorylation of one or more of the five N-terminal serine residues, then mutation of the serine(s) that is (are) phosphorylated should lead to an effect similar to that of the truncation. Since it is not known which of the serines are phosphorylated, we simultaneously mutated all five serines to alanine in the full-length FLAG construct (FLAG-S/A-DAT). To obtain further evidence that phosphorylation of one or more of the N-terminal serines is essential for AMPH-induced DA efflux, we also created a construct in which all five of these serines were simultaneously mutated to aspartate (FLAG-S/D-DAT), in an attempt to simulate phosphorylation of the serines.

Neither the K_m_ nor the V_max_ for tyramine uptake was significantly different in FLAG-S/A-DAT and FLAG-S/D-DAT (see Table 1). The small, nonsignificant reduction in uptake by FLAG-S/A-DAT was accounted for by a similarly decreased number of DAT molecules at the cell surface (see Table 1), suggesting that the turnover rate of the transporter was the same in these two mutants. The apparent K_i_'s for inhibition of tyramine uptake in FLAG-S/A-DAT and FLAG-S/D-DAT by AMPH (41 ± 13 nM and 48 ± 7 nM, respectively; *n =* 3) or by cocaine (331 ± 46 nM and 444 ± 47 nM, respectively; *n =* 4) were not significantly different.

**Figure 3:**
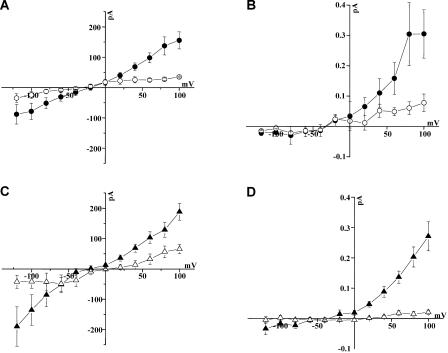
AMPH-Induced Current-Voltage and Amperometric-Voltage Relationships Obtained from FLAG-DAT, FLAG-del22-DAT, FLAG-S/A-DAT, and FLAG-S/D-DAT (A) Current-voltage relationships of AMPH-induced current obtained from FLAG-DAT (filled circles) and FLAG-del22-DAT (open circles) cells. AMPH (10 μM) was applied to the bath while the membrane potential was stepped from–120 mV to +100 mV from a holding potential of –20 mV in 20 mV increments (*n* = 5). (B) Amperometric-voltage relationships obtained from FLAG-DAT (filled circles) and FLAG-del22-DAT (open circles) cells acquired concomitantly to the whole-cell current of panel A. (C and D) Current-voltage (C) and amperometric-voltage (D) relationships of whole-cell and oxidative currents obtained from FLAG-S/D-DAT (filled triangles) and FLAG-S/A-DAT (open triangles) cells using the same experimental protocol as above.

Current-voltage and amperometric-voltage relationships were generated for FLAG-DAT, FLAG-del22-DAT, FLAG-S/A-DAT, and FLAG-S/D-DAT by stepping the voltage from a holding potential of −20 mV to voltages between –120 mV and +100 mV in increments of 20 mV (Figure 3). In FLAG-DAT cells, AMPH-induced currents and DA efflux were voltage dependent, with an increase at positive voltages and saturation of DA efflux near +100 mV (Figure 3A and 3B, filled circles). In contrast, in FLAG-del22-DAT cells, the AMPH-induced currents and DA efflux were greatly reduced at all voltages tested (compare Figure 3A and 3B, open circles, with Figure 3A and 3B, filled circles). This phenomenon was not likely a consequence of an alteration of ion gradients or accumulation of intracellular AMPH, because no significant differences were found between the reversal potentials of the current obtained from FLAG-DAT cells (24.5 ± 5.3 mV) and FLAG-del22-DAT cells (32.6 ± 6.3 mV). In FLAG-DAT cells, the amperometric current at +80 mV was 0.305 ± 0.079 pA (mean ± SEM; *n =* 6) (Figure 3B, filled circles). In contrast, in FLAG-del22-DAT cells the amperometric current recorded at the same potential was significantly reduced (0.077 ± 0.028 pA, mean ± SEM; *p* < 0.05 by Student's t-test, FLAG-del22-DAT versus FLAG-DAT; *n =* 5) (Figure 3B, open circles).

Similarly, in FLAG-S/D-DAT cells the AMPH-induced currents and DA efflux were much greater than those generated in FLAG-S/A-DAT cells (Figure 3C and 3D, filled triangles and open triangles, respectively). In FLAG-S/D-DAT cells, the amperometric current at +80 mV was 0.202 ± 0.039 pA (mean ± SEM; *n =* 7) (Figure 3D, filled triangles). In contrast, in FLAG-S/A-DAT cells, the amperometric current recorded at the same potential was significantly reduced (0.014 ± 0.009 pA, mean ± SEM; *p* < 0.05 by Student's t-test, FLAG-S/D-DAT versus FLAG-S/A-DAT; *n =* 5). Thus, the ability of AMPH to induce DAT-mediated currents and DA efflux was impaired dramatically, either by N-terminal truncation, or by substitution of the five N-terminal serines to alanine. Remarkably, substituting these five serines to aspartate to mimic phosphorylation restored the ability of AMPH to induce voltage-dependent DA efflux and to produce currents, indicating that negative charges in the DAT N-terminal region are essential for these actions of AMPH.

To explore which serine or serines are critical to the effect on efflux, we created five additional mutants in the FLAG-S/A-DAT background in which we mutated each of the five positions, one at time, to aspartate, and we created stable pools of EM4 cells expressing each of these mutants. At +100 mV the amperometric currents in FLAG-S/A-DAT, FLAG-S/A-2D-DAT, FLAG-S/A-4D-DAT, and FLAG-S/A-13D-DAT were 7.4% ± 2.6%, 8.4% ± 5.7%, 11.2% ± 3.1%, and 12.3% ± 7.0%, respectively, of that seen in FLAG-S/D-DAT (*n* = 3; not significantly different from FLAG-S/A-DAT by One-way ANOVA and Tukey's Multiple Comparison Test). In contrast, amperometric currents in FLAG-S/A-7D-DAT and FLAG-S/A-12D-DAT were 29.8% ± 12.6% and 45.1% ± 9.6%, respectively, of that seen in FLAG-S/D-DAT (*n* = 3; *p* < 0.01 compared to FLAG-S/A-DAT by One-way ANOVA and Tukey's Multiple Comparison Test). Thus, negative charge at either position 7 or position 12 restores a substantial fraction of the efflux seen with aspartate at all five positions, and the size of the resulting efflux relative to FLAG-S/D-DAT and FLAG-DAT suggests that both of these serines may be phosphorylated in vivo (see below).

The differences in AMPH-induced DA efflux between FLAG-S/A-DAT and FLAG-S/D-DAT could result either from an altered affinity of DAT for intracellular DA or from a change in the V_max_ of the transport process. At +80 mV, at what is a saturating concentration of intracellular Na^+^ for FLAG-DAT (see “Materials and Methods”), the K_m_ for intracellular DA was 1.4 ± 0.4 mM for FLAG-S/A-DAT and 1.3 ± 0.4 mM for FLAG-S/D-DAT. Thus, a change in the V_max_ of the AMPH-induced DAT-mediated efflux is likely responsible for the differences between FLAG-S/A-DAT and FLAG-S/D-DAT.

Our results suggest that phosphorylation of one or more serines in the N-terminus of the human DAT shifts DAT from a “reluctant” state to a “willing” state for AMPH-induced DA efflux. (A related phenomenon has been proposed for calcium channel regulation [Zhu and Ikeda 1994].) That DAT is significantly phosphorylated under basal conditions and that this phosphorylation can be increased by AMPH (Roxanne Vaughan, pers. comm.) are also consistent with a role for N-terminal phosphorylation in the AMPH-induced efflux mechanism. The structural basis for this regulation of efflux is currently unknown. It may result from a shift in the voltage or sodium dependence of efflux and thus from an increase in the fraction of DAT molecules that reorient to the external milieu empty of DA. Whatever the mechanism, under unclamped, “physiological” conditions, N-terminal phosphorylation does not alter significantly any rate-limiting steps for uptake.

Despite our demonstration that the V_max_ for uptake is unaltered in the mutants, it is possible that phosphorylation might alter the ionic coupling of DAT. The ratio of whole-cell to amperometric current (Galli et al. 1997) at +100 mV was not different in FLAG-DAT and FLAG-del22-DAT (728 ±193 [*n* = 8] and 835 ± 300 [*n* = 5], respectively; *p* > 0.05 by Student's t test). (Similar results were obtained at +60 and +80 mV [data not shown].) This ratio is a microscopic property of an individual transporter that is inversely proportional to the fraction of charge carried by dopamine (Galli et al. 1997). These data, measured in the presence of saturating intracellular dopamine concentrations in the patch pipette, are consistent, therefore, with a similar ionic coupling in the two mutants. However, given the lack of stoichiometric coupling between substrate flux and charge movement (see below), we cannot absolutely rule out an effect of phosphorylation on the ionic coupling of flux. To rule out such a change, it would be helpful to demonstrate that the WT and mutant transporters can generate similar concentration gradients at equilibrium, even though efflux rates differ. In unclamped cells, however, the persistent presence of substrate might lead to changes in membrane potential, and, therefore, such experiments would best be performed under voltage-clamp conditions with an amperometric electrode inside the cell to measure the accumulation of dopamine (Mosharov et al. 2003).

Curiously, AMPH-induced currents, which are largely an uncoupled chloride conductance mediated by DAT that is gated by substrates such as AMPH (Ingram et al. 2002), were reduced in the absence of N-terminal phosphorylation in parallel with DA efflux. Although the underlying mechanisms are unclear, these findings are consistent with the findings of Sitte et al. (1998) that there is a poor correlation between substrate-induced efflux and the uptake of substrates, but a good correlation between the ability of substrates to induce currents and their ability to cause efflux (Khoshbouei et al. 2003).

Regardless of the mechanisms, our findings argue that the mechanism of DA efflux is to some extent independent from the inward-transport process. Since truncation of the N-terminus had the same functional effect as neutralization of the N-terminal serines, it is likely that an essential interaction of the phosphorylated N-terminus of DAT must occur to permit efflux, either with another part of DAT or conceivably with an associated protein. These results could lead to the design and synthesis of new therapeutic agents, such as a drug that blocks the effects of AMPH-like psychostimulants without inhibiting DA uptake. Selective enhancement of DA release might be achieved by promoting phosphorylation of the N-terminus of DAT or by modulating critical interactions of the DAT N-terminus. Furthermore, a polymorphism or naturally occurring mutation of the N-terminal portion of DAT could alter efflux in the context of normal uptake, and this might be associated with human psychiatric or neurologic dysfunction, much as a polymorphism of the norepinephrine transporter has been found to be associated with orthostatic intolerance (Robertson et al. 2001).

Mutations of Ser7 and Ser12 of DAT were found previously to affect the response to inhibition of PKC and MEK1/2, respectively (Lin et al. 2003). We found that negative charge at either of these positions, but not at the positions of the three other N-terminal serines at positions 2, 4, and 13, restored significant AMPH-induced DA efflux. Nonetheless, the serines that are actually phosphorylated as a result of activation of PKC or by AMPH have not been identified, and the kinase or kinases that directly phosphorylate the N-terminus of DAT are unknown as well. Efforts are underway to identify directly the serines that are phosphorylated in vivo, as well as the responsible kinase, and to further uncover the mechanism by which the phosphorylated N-terminus makes DAT “willing” to efflux DA.

## Materials and Methods

### 

#### Plasmid construction, transfection, and cell culture

The N-terminally FLAG-tagged full-length synthetic human DAT (synDAT) gene in pCIHyg was described previously (Saunders et al. 2000). In the FLAG-HA-DAT construct, an HA tag followed the FLAG tag and the first 22 amino acids (MSKSKCSVGLMSSVVAPAKEPN) of human DAT were deleted (Hastrup et al. 2001). In FLAG-del22-DAT, these 22 amino acids were deleted from the full-length FLAG-DAT, making this construct identical to FLAG-HA-DAT except for the absence of the HA-tag sequence. From the FLAG-DAT background, Ser2, Ser4, Ser7, Ser12, and Ser13 were simultaneously mutated to alanine to create the FLAG-S/A-DAT construct and to aspartate to create the FLAG-S/D-DAT construct. The mutant constructs were generated, confirmed, and expressed stably in human embryonic kidney cells (HEK-293) or EM4 cells, HEK-293 cells stably transfected with macrophage scavenger receptor to promote adherence (Robbins and Horlick 1998), as described previously (Hastrup et al. 2001).

#### Uptake of [^3^H]tyramine

Uptake assays with adherent EM4 cells stably expressing the appropriate DAT construct were performed as described previously (Hastrup et al. 2001). Tyramine was used as a radiolabeled substrate because it is not a substrate for catechol-O-methyl transferase, which is endogenously present in HEK-293 cells and EM4 cells, and therefore is not subject to degradation that might complicate the kinetics of uptake (Hastrup et al. 2001). Nonspecific uptake was determined in the presence of 2 mM tyramine. For determination of V_max_ and K_m_ values, increasing concentrations of tyramine from 0.02 to 50 μM were used. K_m_ and V_max_ values for [^3^H]tyramine and [^3^H]dopamine uptake were determined by nonlinear regression analysis using GraphPad Prism 4. IC_50_ values were determined using increasing concentrations of AMPH between 0.002 and 2 μM and of cocaine between 0.001 and 10 μM in competition with approximately 60 nM [^3^H]tyramine. K_i_ values were calculated from the IC_50_ values as described by Cheng and Prusoff (1973).

#### Cell-surface biotinylation and immunoblotting

EM4 cells stably expressing the DAT constructs were incubated with cleavable sulfo-NHS-S-S-biotin (Pierce Chemical Company, Rockford, Illinois, United States) to label surface-localized transporter, and the biotinylated material was prepared and immunoblotted as described previously (Saunders et al. 2000).

#### AMPH-induced DA efflux

Confluent 100-mm plates of HEK-293 cells stably expressing FLAG-DAT or FLAG-HA-DAT were washed twice with KRH (25 mM HEPES [pH 7.4], 125 mM NaCl, 4.8 mM KCl, 1.2 mM KH_2_PO_4_, 1.3 mM CaCl_2_, 1.2 mM MgSO_4,_ and 5.6 mM glucose) and incubated at 37^o^C with 15 μM DA for 30 min. Following incubation, cells were washed with KRH, harvested, resuspended in 0.20 ml of KRH and superfused in a Brandel superfusion apparatus (Brandel SF-12, Gaithersburg, Maryland, United States) as described by Kantor et al. (2001). The KRH contained 10 μM pargyline, and AMPH was added at concentrations from 1 to 100 μM for 2.5 min only. DA was determined by HPLC with electrochemical detection as described by Kantor et al. (2001).

#### Electrophysiology and amperometry

Whole-cell and amperometric currents were recorded as described previously (Khoshbouei et al. 2003). The AMPH-induced whole-cell and amperometric currents were defined as the current recorded in the presence of AMPH, minus the current recorded after the addition of cocaine to the bath with AMPH still present. Previously, we demonstrated that AMPH increases intracellular sodium and that a high concentration of NaCl in the recording pipette maximizes DA efflux (Khoshbouei et al. 2003). Thus, to increase the basal and AMPH-induced DA efflux and to maintain a constant sodium concentration, the whole-cell electrode was filled with internal solution containing 2 mM DA and 90 mM NaCl substituted with KCl to maintain a constant osmolarity of 270 mOsm. The dependence of DA efflux on internal DA was determined by fitting the values of the steady-state amperometric currents, recorded at different intracellular DA concentrations (between 500 μM and 4 mM), to a Hill equation by nonlinear regression. The ratio of whole-cell to amperometric current was calculated by dividing the average whole-cell current during the last 100 ms of the voltage step by the average amperometric current during the same time period (Galli et al. 1998).

## Supporting Information

Accession Numbers

The Swiss-Prot (http://ca.expasy.org/cgi-bin/niceprot.pl?Q01959) entry name for the gene discussed in this paper is S6A3_HUMAN, accession number Q01959.

## Abbreviations

AMPH: amphetamine
DA: dopamine
DAT: dopamine transporter
PKC: protein kinase C
WT: wild-type
